# Evaluation of Alternative Transport Media for RT-qPCR-Based SARS-CoV-2 Testing

**DOI:** 10.1155/2022/5020255

**Published:** 2022-08-10

**Authors:** Young Hyun Baek, Min Young Park, Ho Jae Lim, Hye Soo Jung, Jae-Hyun Yang, Yong-Hak Sohn, Sun-Hwa Lee, Jung Eun Park, Yong-Jin Yang

**Affiliations:** ^1^Department of Molecular Diagnostics, Seegene Medical Foundation, Seoul 04805, Republic of Korea; ^2^Department of Integrative Biological Sciences & BK21 FOUR Educational Research Group for Age-associated Disorder Control Technology, Chosun University, Gwangju 61452, Republic of Korea; ^3^Paul F. Glenn Center for Biology of Aging Research, Department of Genetics, Blavatnik Institute, Harvard Medical School, Boston, MA 02115, USA; ^4^Department of Laboratory Medicine, Seegene Medical Foundation, Seoul 04805, Republic of Korea

## Abstract

Severe acute respiratory syndrome coronavirus 2 **(**SARS-CoV-2), which causes coronavirus disease 2019 (COVID-19), is still rapidly spreading as of March 2022. An accurate and rapid molecular diagnosis is essential to determine the exact number of confirmed cases. Currently, the viral transport medium (VTM) required for testing is in short supply due to a sharp increase in the laboratory tests performed, and alternative VTMs are needed to alleviate the shortage. Guanidine thiocyanate-based media reportedly inactivate SARS-CoV-2 and are compatible with quantitative reverse transcription polymerase chain reaction (RT-qPCR) assays, but the compatibility and the viral detection capacity have not been fully validated. To evaluate the guanidine thiocyanate-based Gene Transport Medium (GeneTM) as an alternative VTM, we prepared 39 SARS-CoV-2-positive and 7 SARS-CoV-2-negative samples in GeneTM, eNAT™, and phosphate-buffered saline (PBS). The cycle threshold (Ct) values of three SARS-CoV-2 targets (the S, RdRP, and N genes) were analyzed using RT-qPCR testing. The comparison of Ct values from the positive samples showed a high correlation (*R*^2^= 0.95–0.96) between GeneTM and eNAT™, indicating a comparable viral detection capacity. The delta Ct values of the SARS-CoV-2 genes in each transport medium were maintained for 14 days at cold (4°C) or room (25°C) temperatures, suggesting viral samples were stably preserved in the transport media for 14 days. Together, GeneTM is a potential alternative VTM with comparable RT-qPCR performance and stability to those of standard media.

## 1. Introduction

Coronavirus disease 2019 (COVID-19), caused by severe acute respiratory syndrome coronavirus 2 (SARS-CoV-2), was first reported in December 2019 in Wuhan, China [1]. Owing to its highly infectious nature, COVID-19 has spread rapidly worldwide [2, 3]. Precautionary measures (such as physical isolation, social distancing, and the use of face masks) and an accurate and rapid diagnosis of COVID-19 are important preventive measures for controlling its spread [4].

The Centers for Disease Control and Prevention (CDC) of the United States uses viral and antibody tests to diagnose COVID-19. Viral tests include the nucleic acid amplification test (NAAT) and the antigen test, which allow the diagnosis of the current infection status; on the other hand, antibody tests help in the detection of a past infection. The NAAT is designed to detect the genetic material (RNA) of the virus through molecular methods, such as quantitative reverse transcription polymerase chain reaction (RT-qPCR) and loop-mediated isothermal amplification. Conversely, the antigen test is designed to detect viral antigens on the surface of SARS-CoV-2 [5]. The NAAT has the advantage of being the most sensitive diagnostic test available for confirming the current state of infection [6, 7]. The primary specimens used for RT-qPCR testing include nasopharyngeal swabs (NPS) and oropharyngeal swabs. The swabs are immersed immediately in a viral transport medium (VTM), which preserves the viral viability and supports molecular diagnostics [8]. Therefore, the safety of the sample in the VTM is very important for accurate diagnosis.

Recently, the number of confirmed COVID-19 cases has increased significantly due to the highly infectious nature of SARS-CoV-2 [9, 10]. Its rapid spread within the population has significantly increased the need for laboratory testing, which has led to a shortage of the universal VTM for nasopharyngeal sampling [11]. It may be possible to resolve this issue with alternative VTMs that are safe and suitable for diagnostic tests.

Saline and phosphate-buffered saline (PBS) are VTM materials recommended by the U.S. Food and Drug Administration (FDA) for SARS-CoV-2 testing and COVID-19 diagnostic testing [12]. Clinical specimens for SARS-CoV-2 testing may be exposed to biological risks during transportation to the testing lab or during test processing [13]. Furthermore, several of the physical and chemical methods that are used for viral inactivation or RNA extraction are not suitable for designing safe transport media [14–17], and additional requirements can complicate rapid COVID-19 testing [18]. There are studies on guanidine thiocyanate-based media that are suitable for virus inactivation and RT-qPCR detection assays [19]. eNAT™ (Copan, Brescia, Italy) is a guanidine thiocyanate-based medium that can help nucleic acids remain stable for a long period and is suitable for sample collection and transport [20]. Similarly, GeneTM is a guanidine thiocyanate-based medium that is suitable for collecting nasal (nasopharyngeal) and oral (oropharyngeal or salivary) samples for respiratory infection testing; it can also be used for the transportation and preservation of samples for COVID-19 testing.

For this reason, we compared GeneTM and eNAT™ (with PBS as a control) to determine their suitability as appropriate transport media using confirmed SARS-CoV-2-positive and SARS-CoV-2-negative samples. In addition, we compared the three media to evaluate the stability of incubation conditions and storage duration.

## 2. Materials and Methods

### 2.1. Clinical Specimens

Anonymized residual NPS specimens in the clinical transport medium (CTM; Noble Biosciences, Hwaseong, Republic of Korea) were preserved in April 2021 as either SARS-CoV-2-positive samples (*n* = 39) or SARS-CoV-2-negative samples (*n* = 7). All SARS-CoV-2-positive samples had high viral copy numbers.

### 2.2. Sample Preparation and Viral RNA Extraction

**Figure 1:**
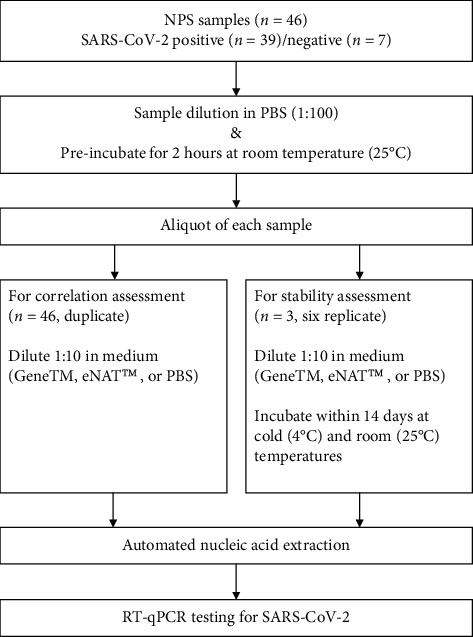
Assessment flowchart of the alternative transport media for SARS-CoV-2 testing. NPS, nasopharyngeal swab; PBS, phosphate-buffered saline; GeneTM, Gene transport medium; RT-qPCR, quantitative reverse transcription polymerase chain reaction.

The following two VTMs were used for SARS-CoV-2: eNAT™ (Copan, Brescia, Italy) and GeneTM (SG Medical, Seoul, Republic of Korea). PBS (Biosesang Co., Seongnam, Republic of Korea) was used as a control. All CTM samples were diluted to 1:100 in PBS and incubated for 2 hours. A 50 *μ*L aliquot of each specimen (diluted 1:10) was added to 450 *μ*L of each transport medium (GeneTM, eNAT™, and PBS). Overall, 46 samples were used for correlation tests from two vials of each transport medium. Three SARS-CoV-2-positive samples were selected for stability assessment, and 72 vials of additional samples were prepared in each medium. All samples were processed using an automated nucleic acid extraction system (MagNA Pure 96; Roche, Basel, Switzerland), in accordance with the “Pathogen Universal 200” protocol described in a previous study [21]. In brief, the MagNA Pure 96 DNA and Viral NA Small Volume kit (Roche) was used, and 200 *μ*L of each sample was transferred to the cartridge. The main processing steps of this study are summarized in Figure 1.

### 2.3. Multiplex RT-qPCR Analysis

SARS-CoV-2 RNA was subjected to molecular analysis using the Allplex™ SARS-CoV-2/FluA/FluB/RSV assay (Seegene, Seoul, Republic of Korea) in accordance with the manufacturer's instructions [22]. The genes for the SARS-CoV-2 spike (S), RNA-dependent RNA polymerase (RdRP), and nucleocapsid protein (N) were detected. These assays were performed using CFX96™ (Bio-Rad Laboratories, Hercules, CA, USA). The amplification conditions were as follows: 50°C for 20 min; 95°C for 15 min; three cycles of 95°C for 10 s, 60°C for 40 s, and 72°C for 20 s for preamplification; and 42 cycles of 95°C for 10 s, 60°C for 15 s, and 72°C for 10 s. Data were analyzed using the Seegene Viewer for Real-time Instruments v3.24 (Seegene, Seoul, Republic of Korea); a positive result was considered if more than one Ct value was under 35, regardless of the results of the internal control [23].

### 2.4. Correlation Assessment and Stability Assessment

Together with the cycle threshold (Ct) values of SARS-CoV-2 samples, the correlations were tested before storage (0 day) to obtain the initial Ct value. For intraassay (intrasample), 39 positive samples were analyzed, while for interassay (intersample), all samples (39 positive, 7 negative) in this study were used, and negative samples were considered if they had the Ct value as 41. The stability tests, sample with low Ct values (Ct < 26), were performed at multiple time points for up to 14 days (i.e., at 1, 2, 4, 7, 10, and 14 days) and under two storage conditions: cold temperature (4°C) and room temperature (25°C). Six replicates were assayed for each condition. In order to analyze the relative changes in the Ct value, the delta Ct method (elapsed days–day 1) was selected for stability assessment. Samples were deemed stable if the mean Ct values did not increase by more than 3 amplification cycles of the delta Ct value [24].

### 2.5. Data Analysis

The boxplots and time series plots were illustrated using the ggplot2 package in *R* studio (version 4.1.2; *R* studio, Boston, Massachusetts, USA). Scatterplot, one-way analysis of variance (ANOVA), and Pearson correlation analyses were performed to quantify the associations using SPSS (version 26.0; IBM Corp., Armonk, NY, USA) for Mac. Two replicated samples were averaged within the subject, the transport media, and the three genes. The intraassay of boxplots was determined for the correlation in each transport medium. The interassay of scatter plots included the diagonal line (slope = 1) to show that the difference between the two transport media is skewed to one side. These tests were performed separately among the transport media for each of the three genes. Both correlation tests were performed for statistical significance by ANOVA. The *P* value < 0.05 was considered significant [25]. To determine the stability of SARS-CoV-2, relevant data were analyzed using the ggplot2 package in *R* studio. The average and standard error of data from the six replicates were calculated following delta Ct values of 3 genes from each transport medium. For six replicates, the coefficient of variance (CV%) < 7% was considered acceptable for the variability of the replicates [26].

## 3. Results

### 3.1. Compatibility of GeneTM with Each Transport Medium

**Figure 2:**
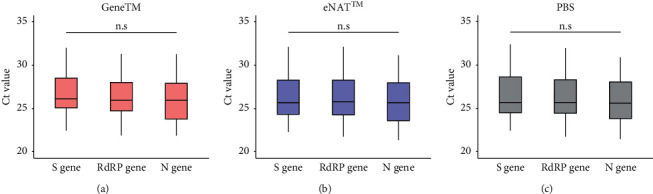
Distribution of Ct values for matched SARS-CoV-2-positive NPS samples and the three SARS-CoV-2 genes S, RdRP, and N in the three transport media. (a) GeneTM. (b) eNAT™. (c) PBS. Two replicates per sample were assayed using real-time PCR under the indicated conditions. PBS, phosphate-buffered saline; Ct, cycle threshold; S gene, spike gene; RdRP gene, RNA-dependent RNA polymerase gene; N gene, nucleocapsid gene; n.s., not significant.

Boxplots show the distribution of the Ct value, obtained from the 39 SARS-CoV-2-positive samples in each transport medium (Figure 2). The effects of each medium on the diagnostic accuracy and Ct value of the SARS-CoV-2 sample were evaluated. All the 39 known positive samples, diluted in each transport medium, were positive for the virus, with Ct values ranging from 21 to 33. Assessment of the intraassay variability showed that none of the 39 positive specimens showed significant differences in Ct values for the SARS-CoV-2 gene S, RdRP, and N. These findings confirmed that GeneTM does not affect the detection of SARS-CoV-2.

### 3.2. Comparison of Ct Values of GeneTM, eNAT™, and PBS

**Figure 3:**
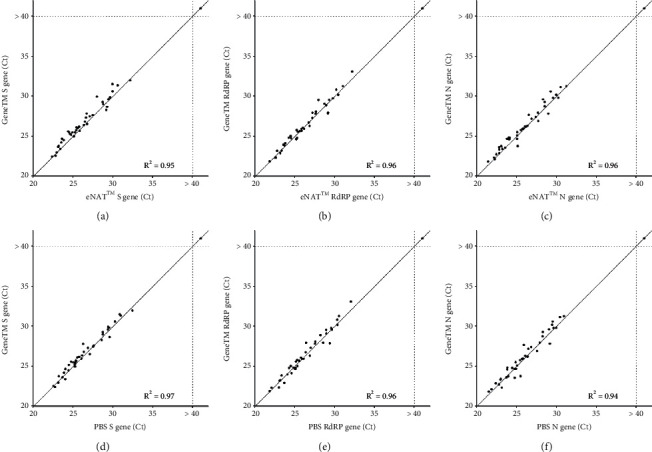
Comparison of the Ct values (determined by real-time PCR) for the three SARS-CoV-2 genes in the 46 NPS samples that were positive or negative for the virus. Samples were diluted in GeneTM, eNAT™, or PBS. Comparison of the (a) S gene, (b) RdRP gene, and (c) N gene in NPS samples diluted in GeneTM and eNAT™. Comparison of the (d) S gene, (e) RdRP gene, and (f) N gene in NPS samples diluted in GeneTM and PBS. Two replicates per sample were assayed using real-time PCR under the indicated conditions. Ct, cycle threshold; S gene, spike gene; RdRP gene, RNA-dependent RNA polymerase gene; N gene, nucleocapsid gene.

To evaluate the correlation of Ct values from samples in different media, Ct values of GeneTM were compared to those of eNAT™ or PBS. The correlation (*R*^2^) and the difference of Ct values between GeneTM and eNAT™ were 0.95–0.96 and 0.14–0.46, respectively (Figures 3(a)–3(c)). Between GeneTM and PBS, the correlation (*R*^2^) value was between 0.94 and 0.97; the difference in the Ct value was between 0.15 and 0.25 (Figures 3(d)–3(f)). These findings showed that the Ct values of the three genes were not substantially observed by the dilution medium, indicating that GeneTM had no effect on gene abundance in comparison with eNAT™ or PBS.

### 3.3. Stability of SARS-CoV-2 RNA in the Transport Media

**Figure 4:**
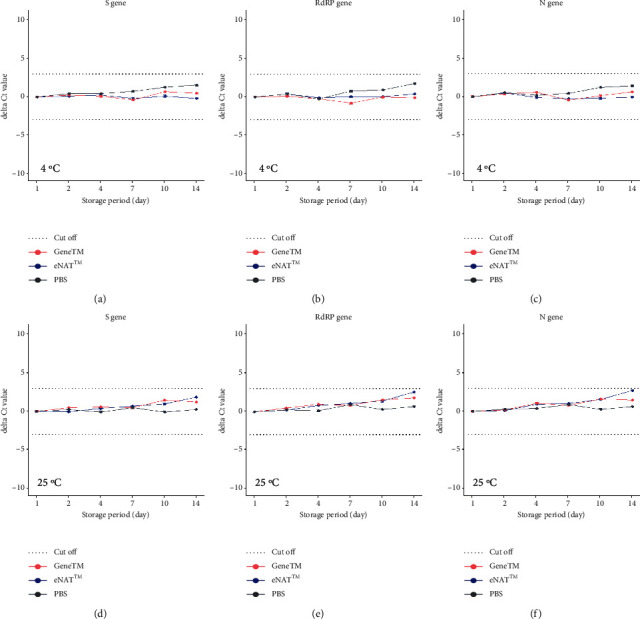
Stability of the SARS-CoV-2 (a, d) S gene, (b, e) RdRP gene, and (c, f) N gene following dilution of the NPS samples in CTM in GeneTM, eNAT™, or PBS at (a–c) cold temperature (4°C) and (d–f) room temperature (25°C). Six replicates per sample were assayed by real-time PCR under each of the indicated conditions. The dotted line indicated the cut-off value of ±3 Ct, below which samples were deemed stable. Ct, cycle threshold; S gene, spike gene; RdRP gene, RNA-dependent RNA polymerase gene; N gene, nucleocapsid gene.

All results represent the mean of six replicates stored under each condition with one SARS-CoV-2-positive NPS sample. Samples stored at a cold temperature in GeneTM, eNAT™, and PBS showed differences in the delta Ct values, measured as −0.24 to 0.50, −0.79 to 0.66, and −0.23 to 1.76, respectively, depending on the SARS-CoV-2 gene assayed (Figures 4(a)–4(c)). Similarly, samples stored at room temperature in GeneTM, eNAT™, or PBS showed differences in delta Ct values, measured as 0 to 1.81, −0.06 to 2.69, and −0.10 to 0.94, respectively, depending on the SARS-CoV-2 gene assayed (Figures 4(d)–4(f)). When another SARS-CoV-2-positive sample was added to the media and analyzed under the same conditions, same results were obtained (Supplementary Figure 1). Overall, the CV values were measured as less than 4.5%, indicating acceptable variability (Supplementary Table 1). These results revealed the stable maintenance of each SARS-CoV-2 gene for 14 days in each transport medium, regardless of the storage temperature.

## 4. Discussion

As the number of COVID-19 cases continues to increase, more testing is required to determine the exact number of people infected. Molecular diagnostic methods are crucial for obtaining accurate and timely data that influence public health policy decisions [27]. NAATs are the most sensitive diagnostic tests available and usually do not need to be repeated to confirm the results [7]. Specimens used primarily for RT-qPCR testing include NPS and oropharyngeal swabs stored in the VTM. However, the high demand for testing has led to a shortage of the VTM required for RT-qPCR testing of SARS-CoV-2 [11, 28, 29]. Thus, during the COVID-19 pandemic, the FDA allowed laboratories to consider the use of alternative transport media [12]. Among these, the guanidine thiocyanate-based eNAT™ medium reportedly inactivates SARS-CoV-2 and stabilizes its nucleic acid [19, 20, 30]. In this study, we demonstrated the efficacy of GeneTM (another guanidine thiocyanate-based medium) by comparing it with eNAT™, using PBS as a control.

We found that GeneTM showed high intrasample and intersample reliabilities in RT-qPCR assays of SARS-CoV-2 RNA (Figures 2 and 3), and both showed no statistically significant difference in correlation test, respectively (*P* > 0.05). GeneTM was equivalent to eNAT™ when assessing the viral load in NPS samples stored for up to 14 days at room and cold temperatures (Figure 4, Supplementary Figure 1). Stability assessment did not show any significant effects in these genes by storage temperature, time, or transport medium. Ct values of the positive samples in GeneTM with unknown viral loads showed a positive correlation with those of the same samples in either PBS or eNAT™, indicating that GeneTM is a dependable transport medium for use with clinical samples.

The results of this analysis are consistent with those showing that GeneTM is equivalent to eNAT™ and PBS when known quantities of SARS-CoV-2 are added to each medium. There was little decay in the signal after storage times of up to 14 days. We also focused on three SARS-CoV-2 genes across several samples, supporting the robustness of the entire process (including sample transport). Furthermore, sample processing in the laboratory is often delayed in busy clinical settings; accordingly, GeneTM is advantageous because it acts as a stable storage medium and prevents significant viral decay for up to 14 days at room temperature before RT-qPCR (Figure 4, Supplementary Figure 1).

A limitation of this study was that the NPS samples in CTM were spiked into each medium. The amount of sample obtained was not sufficient to match the requirement of the experiment; thus, the experiment was conducted by spiking GeneTM, eNAT™, and PBS using diluted samples with high viral load (low Ct value). Therefore, further studies are needed to evaluate equivalence in low viral load samples.

The stability of SARS-CoV-2 in the environment, which contributes to its widespread dissemination, may eliminate the need for rapid transport of clinical specimens. The extent to which clinical laboratories can respond to the COVID-19 pandemic is linked to their ability to develop and deploy proper diagnostic procedures. Early detection of SARS-CoV-2 allows prompt treatment of the infected patients and a rapid implementation of control measures to limit viral transmission. Expanded testing capabilities would also facilitate widespread surveillance and infection containment in communities, which could support policies for reducing restrictions on work, travel, and social distancing.

## 5. Conclusions

The current study indicates that GeneTM is a clinically useful transport medium with the potential to increase the detection capacity for SARS-CoV-2, thereby improving surveillance and clinical care.

## Data Availability

The data used to support the findings of this study are included within the article.

## Supplementary Materials

Supplementary Materials Supplementary Figure 1: (a–c) stability of SARS-CoV-2 genes at cold temperature (4°C). (d–f) Stability of SARS-CoV-2 genes at room temperature (25°C). (a, d) S gene. (b, e) RdRP gene. (c, f) N gene. Two NPS samples in CTM diluted in GeneTM, eNAT™, and PBS. Six replicates per samples were assayed by real-time PCR under each of the indicated conditions. The dotted line indicated the cut-off value of ±3 Ct, below which samples were deemed stable. S gene, spike gene; RdRP gene, RNA-dependent RNA polymerase gene; N gene, nucleocapsid gene. Supplementary Table 1: coefficient of variation (CV, in %) for storage durations and incubation conditions in the viral transport medium. Data are presented as the minimum and maximum coefficient of variance during 14 days in stability tests of three samples. Abbreviations: S gene, spike gene; RdRP gene, RNA-dependent RNA polymerase gene; N gene, nucleocapsid gene.
