# Supplementary material for: Evaluation of Alternative Transport Media for RT-qPCR-Based SARS-CoV-2 Testing
Source: Int J Anal Chem. 2022 Aug 10;2022:5020255. doi: 10.1155/2022/5020255 (PMC9385321; doi:10.1155/2022/5020255)
Supplement: Supplementary Materials — Supplementary Figure 1: (a–c) stability of SARS-CoV-2 genes at cold temperature (4°C). (d–f) Stability of SARS-CoV-2 genes at room temperature (25°C). (a, d) S gene. (b, e) RdRP gene. (c, f) N gene. Two NPS samples in CTM diluted in GeneTM, eNAT™, and PBS. Six replicates per samples were assayed by real-time PCR under each of the indicated conditions. The dotted line indicated the cut-off value of ±3 Ct, below which samples were deemed stable. S gene, spike gene; RdRP gene, RNA-dependent RNA polymerase gene; N gene, nucleocapsid gene. Supplementary Table 1: coefficient of variation (CV, in %) for storage durations and incubation conditions in the viral transport medium. Data are presented as the minimum and maximum coefficient of variance during 14 days in stability tests of three samples. Abbreviations: S gene, spike gene; RdRP gene, RNA-dependent RNA polymerase gene; N gene, nucleocapsid gene. [file 5020255.f1.zip › 5020255.f1/Supplementary Figure 1 (1).docx]

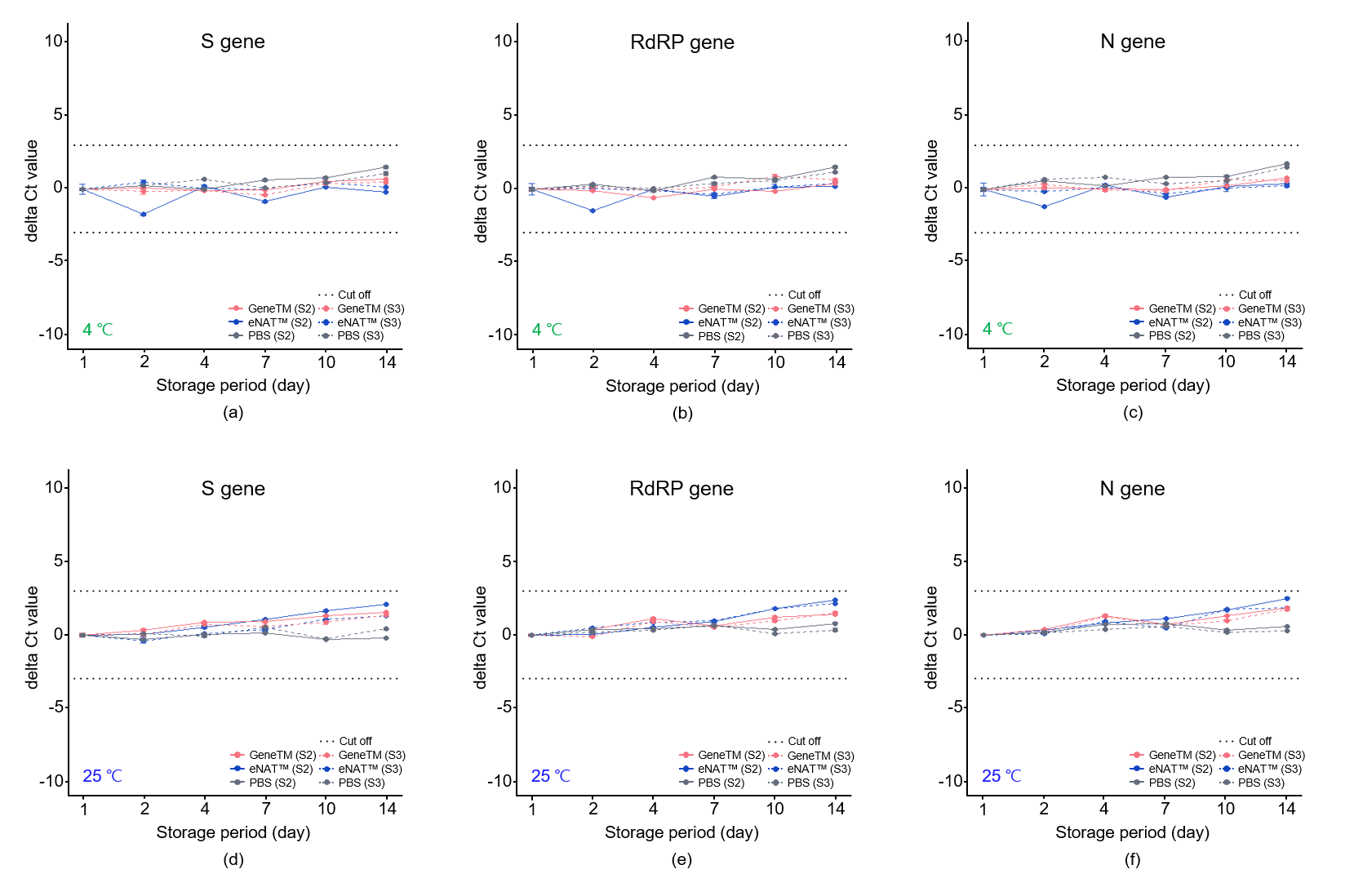


**Supplementary Figure 1:** (a–c) Stability of the SARS-CoV-2 genes at cold temperature (4 ℃). (d–f) Stability of the SARS-CoV-2 genes at room temperature (25 ℃). (a, d) S gene. (b, e) RdRP gene. (c, f) N gene. Two NPS samples in CTM diluted in GeneTM, eNAT^TM^, and PBS. Six replicates per samples were assayed by real-time PCR under each of the indicated conditions. Dotted line indicated cut-off value of ± 3 Ct, below which samples were deemed stable. S gene, spike gene; RdRP gene, RNA-dependent RNA polymerase gene; N gene, nucleocapsid gene.
